# Tracking human genes along the translational continuum

**DOI:** 10.1038/s41525-019-0100-0

**Published:** 2019-10-16

**Authors:** Kyubum Lee, Mindy Clyne, Wei Yu, Zhiyong Lu, Muin J. Khoury

**Affiliations:** 1 National Center for Biotechnology Information (NCBI), National Library of Medicine (NLM), National Institutes of Health (NIH), Bethesda, MD USA; 2 Division of Cancer Control and Population Sciences, National Cancer Institute (NCI), Bethesda, MD USA; 3 Office of Public Health Genomics, Centers for Disease Control and Prevention (CDC), Atlanta, GA USA

**Keywords:** Translational research, Genetics research

## Abstract

Understanding the drivers of research on human genes is a critical component to success of translation efforts of genomics into medicine and public health. Using publicly available curated online databases we sought to identify specific genes that are featured in translational genetic research in comparison to all genomics research publications. Articles in the CDC’s Public Health Genomics and Precision Health Knowledge Base were stratified into studies that have moved beyond basic research to population and clinical epidemiologic studies (T1: clinical and population human genome epidemiology research), and studies that evaluate, implement, and assess impact of genes in clinical and public health areas (T2+: beyond bench to bedside). We examined gene counts and numbers of publications within these phases of translation in comparison to all genes from Medline. We are able to highlight those genes that are moving from basic research to clinical and public health translational research, namely in cancer and a few genetic diseases with high penetrance and clinical actionability. Identifying human genes of translational value is an important step towards determining an evidence-based trajectory of the human genome in clinical and public health practice over time.

## Introduction

In spite of ongoing progress in human genomics, only a few clinical and public health applications have been launched as promised by the Human Genome Project.^1^ In addition to technological challenges centered around the availability of accurate and relatively inexpensive genome sequencing, a major challenge has been the selection of genes for research studies. It has been previously observed that most biomedical research on human genes only concentrates on approximately 2000 genes in the human genome. Stoeger et al.^2^ recently explored explanations for this observation by compiling an extensive resource database, including chemical and biological properties of gene-encoded proteins, and the published scientific literature on individual genes. They used machine learning methods to predict the number of publications on individual genes, the year of the first publication about them, the extent of funding by the National Institutes of Health, and the existence of related medical drugs. They found that biomedical research is primarily guided by the generic chemical and biological characteristics of genes, rather than their relevance to human disease.

Could the choice of genes for basic biomedical research guide or influence further steps along translation to clinical genome applications and public health impact? Previously, we have established and characterized four phases of genomics translation from discovery to population health outcomes (T0: discovery, T1: “bench to bedside”, T2: evaluation, T3: implementation, T4: outcomes and population impact^3^) (Supplementary Fig. 1). We have also documented that most genomic research is discovery-based and very little is published in the later phases of translation (T2–T4).^4–6^

It is important to understand the drivers of research on human genes, what biases exist regarding those which are studied, and conversely, to be able to identify currently unstudied or understudied genes which have the greatest potential for translational research success. Specifically, if advances in genomics are going to have an impact on clinical and public health practice, we need to understand the spectrum of translational research in human genes and why only some genes make it all the way through the translation highway (T4).

We sought to learn the specific genes that have made it further along the translational pathway, first to population and clinical epidemiologic studies (“bench to bedside”: (T1)), and then to the evaluation, implementation, and impact studies (“beyond bench to bedside”: T2–T4), respectively. Building on the work of Stoeger et al.,^2^ through our comparative analysis of genes and their respective publication count within these specified areas of the translational pathway, we hope this work can help to clarify which genes and the characteristics of genes that should receive translational research attention beyond bench to bedside.

## Results

We used the Public Health Genomics and Precision Health Knowledge Base (PHGKB),^7^ a curated suite of genomics databases maintained by the CDC Office of Public Health Genomics which tracks the impact and translation of genome discoveries on clinical practice and public health. We included two databases from PHGKB in this exercise: (1) the Human Genome Epidemiology Navigator (HuGE, https://phgkb.cdc.gov/PHGKB/hNHome.action)^8,9^ is a collection of publications on population and clinical epidemiologic studies of human genes in relation to health outcomes, corresponding with T1 translation and (2) the Genomics & Precision Health Database (GPH, https://phgkb.cdc.gov/PHGKB/translationStartPage.action)^6^ which is a collection of publications reflecting the T2-T4 stages of translation. These two databases were compared to all gene-associated Medline/PubMed publications. The details of the stages T1–T4 are explained in Supplementary Fig. 1 and Supplementary Note 1.

The PubMed articles with associated genes ascertained through the gene2pubmed file contained 609,633 PMIDs. The HuGE database and the subset from GPH of original research studies, studies on evidence synthesis, and/or guidelines publications contained 143,417 and 8526 PMIDs, respectively.

## Number of genes represented in publications from HuGE and GPH

While PubMed articles were associated with 24,656 human genes, HuGE and GPH only identified 11,081 and 1846 genes, respectively (Table 1), representing 44.94% and 7.49% relative to the genes that appeared in PubMed (Supplementary Fig. 2). Most of the genes mentioned in GPH are also in HuGE (*n* = 1682). However, 164 genes (8.88%) in GPH were not mentioned in HuGE. Over 96% of these were associated with a publication count of one (*n* = 158).

**Table 1:** Overall number of genes mentioned and gene-specific publication count categorized by (1) rank of top most common and (2) overall percentage of publications in PubMed, HuGE, and GPH

	PubMed	HuGE	GPH
Total # of genes	24,656	11,081	1846
Top 5 genes	2.16%	7.18%	19.36%
Top 10 genes	3.50%	12.13%	28.31%
Top 20 genes	5.54%	19.54%	37.64%
Top 30 genes	7.25%	24.36%	43.19%
Top 50 genes	10.17%	31.16%	49.14%
Top 100 genes	15.17%	41.83%	57.67%
Top 200 genes	21.56%	53.59%	66.98%
Top 400 genes	29.80%	65.16%	76.86%
Top 500 genes	32.83%	68.76%	79.74%
Top 1000 genes	43.55%	79.54%	88.81%
Top 2000 genes	56.28%	88.79%	100.00% (1846 genes)
Top 1% genes	23.83%	43.54%	36.23%
Top 5% genes	47.21%	70.50%	56.61%
Top 10% genes	60.44%	81.05%	65.84%

The most common genes based on publication count in PubMed, HuGE and GPH are listed in Table 2. The top 10 most common genes are represented in 3.50% of all PubMed publications. The top 10 common genes represented in HuGE and GPH are 12.13% and 28.31%, respectively (Table 1).

**Table 2:** Genes ranked by publication count in PubMed, HuGE, and GPH

	Publication count			Statistical significance of the publications compared to PubMed	
Rank	PubMed	HuGE	GPH	HuGE	GPH
1	TP53	APOE	BRCA1	MTHFR	BRCA1
2	TNF	MTHFR	BRCA2	APOE	BRCA2
3	EGFR	TNF	EGFR	HLA-DRB1	PMS2
4	VEGFA	EGFR	ERBB2	GSTM1	MSH6
5	IL6	HLA-DRB1	KRAS	KRAS	MSH2
6	APOE	TP53	TP53	ACE	LDLR
7	TGFB1	ACE	BRAF	GSTT1	MLH1
8	MTHFR	IL6	MLH1	COMT	ERBB2
9	ESR1	KRAS	MSH2	BRAF	KRAS
10	AKT1	GSTM1	LDLR	IL10	PALB2
11	HIF1A	IL10	MSH6	CYP2C19	BRAF
12	NFKB1	GSTT1	PMS2	VDR	EGFR
13	IL10	COMT	CYP2C19	SLC6A4	NRAS
14	BRCA1	BRAF	PIK3CA	CYP3A5	CYP2C19
15	ERBB2	SLC6A4	CYP2D6	ABCB1	CYP2D6
16	MMP9	BRCA1	NRAS	EGFR	PCSK9
17	HLA-DRB1	ABCB1	ALK	CYP2C9	CHEK2
18	IL1B	VDR	CFTR	GSTP1	ALK
19	ACE	BRCA2	CHEK2	CYP2D6	PIK3CA
20	APP	BDNF	PCSK9	TNF	VKORC1

The ranking of “Publication count” column is simply sorted by the number of appearances in each database, and the other column is calculated and ranked using the *z*-score of each gene representing the significance of the publication count difference compared with PubMed

Some genes are significantly more or less popular in HuGE and GPH than PubMed. Table 2 shows the top 20 most significant genes in HuGE and GPH compared to PubMed.

Nine of the top 10 genes in GPH are cancer-related genes. These genes are associated with hereditary breast and ovarian cancer (BRCA1, BRCA2), Lynch syndrome (MLH1, MSH2, MSH6, PMS2), and her2/neu mutations in breast cancer (ERBB2). LDLR is one gene associated with familial hypercholesterolemia. Nine of the 10 top genes are hereditary single gene disorders.

In contrast, the top 10 genes studied in HuGE include genes relevant to many disease conditions. The top gene is MTHFR, a gene associated with defects in folic acid metabolism extensively studied in relation to birth defects, cancer, cardiovascular disease, and other conditions, but has yet to be “translated” into implementation in practice. Similarly, the APOE gene has been popularized resulting from the strong association of APOE4 alleles with Alzheimer’s disease. APOE variation has been studied in relation to cardiovascular diseases and other outcomes.

## Correlation between the publications in PubMed, HuGE, and GPH databases

Supplementary Figure 3a shows the correlation between all the publications in PubMed and the publications in HuGE. The publication count in HuGE is closely and positively related to the publication count in PubMed (Pearson correlation coefficient is 0.76). We found that all top 20 most published genes in HuGE are included in the top 0.5% of the most published genes in PubMed.

As shown in Supplementary Fig. 3b, the publication count in GPH is also positively correlated to that of PubMed. However, this correlation is weaker than the correlation between HuGE and PubMed results (Pearson correlation coefficient is 0.40). We also observe that BRCA1, BRCA2, or HER2 have significantly more publications in GPH compared to PubMed which are far from the fitted linear regression line. Supplementary Figure 3b also shows that GPH focuses on only a few selected genes. Only 9 out of the top 20 genes in GPH are in the top 0.5% of the most published genes in PubMed. The change by year in the number of publications in each database for BRCA1, APOE, LDLR, GJB2, and EGFR are shown in Supplementary Fig. 4. Most of the HuGE and GPH publications on BRCA1 and EGFR genes are cancer-related. The figure also shows that GJB2 and LDLR publications are mostly describing rare diseases and heart, lung, blood, and sleep (HLBS) disorders, respectively.

## Discussion

Using well established and curated publically available publication databases, we have extended the analysis of Stoeger et al. on the overall publications on human genes to publications in translational phases. Our overall goal is to describe which genes have been more likely to be studied epidemiologically (T1) or evaluated and implemented in clinical and public health practice (T2–T4). We observed that translational studies focus on only a small number of human genes, and the farther along the continuum, the smaller the number.

Stoeger et al.^2^ reported that most of the research focuses on only around 2000 genes in PubMed. In our analysis, we found that epidemiology and translational studies focus on an even much smaller number of genes. First, the number of genes and the number of publications represented in HuGE and GPH are a significantly small proportion of all PubMed articles, and only a limited number of genes are the main focus in epidemiology and translational studies.

It is evident from this analysis through the observation of top genes that the “action” in translation beyond bench to bedside is in the field of cancer, including genes associated with hereditary breast and ovarian cancer and Lynch syndrome. These two hereditary cancers have emerged as conditions with important clinical applications in large part due to the demonstration through clinical and epidemiologic studies of the clinical validity and utility of genetic testing shown to reduce morbidity and mortality from these cancers.^10^ The two conditions are also part of the CDC tier 1 classification schema for genomic implementation in practice.^11^ Briefly, this three-tier classification system was developed by CDC to describe the current status of genomics in practice based on evidence of validity and utility, as well as recommendations by guideline groups such as the US Preventive Service Task Force^12^ and others. Another top gene on the list in GPH database is LDLR which is gene for familial hypercholesterolemia, another common autosomal dominant condition associated with premature heart disease, with evidence of clinical utility for testing patients and relatives, and aggressive treatment with cholesterol-lowering drugs.^13^

The smaller number of genes and publications identified in HuGE and GPH might be expected considering that most research further along the translational continuum requires large-scale clinical and population studies, which could be challenging especially for rare diseases. Only the genes that are thoroughly researched and understood, and genes on which there is a sufficient amount of information are more likely to be used for epidemiology, translational, and implementation studies. Other than single gene disorders, given the complexity of human diseases involving genetic and environmental risk factors, most of the studied genes have not made it to clinical or public health purposes. These include most pharmacogenomic traits, HLA gene, as an example. There is also a direct influence on translational research based on what initial basic discovery work is conducted.

Our study has several strengths and limitations. We were able to use curated and well characterized databases and automated methods to quickly correlate the massive amount of biomedical literature on human genes. In particular, the inherent database linkages between HuGE and GPH allowed us to rapidly characterize the translational trajectory of human genes from discovery to clinical practice. However, this analysis is limited by our inability to utilize full text of articles for the identification of genes, and the potential for missing gene information especially from genome-wide HuGE publications. We also recognize potential errors in extraction of data through the computational tools.

These data and the online databases from which they are derived provide baseline information on translation of human genes are made available to other investigators to conduct analyses on specific genes or classes of genes of interest. Future research in this area should be focused on predictors of translation, utilizing bioinformatics tools and available databases.

Analyses such as ours are inherently limited by the inability to identify a chain of causality in the association between the numbers of early discovery research articles and later translational research publications. It is entirely possible the more genes are studied, the more the likelihood at establishing clinical validity and utility for later translational and implementation studies. It is also unclear what classes of mediating and confounding factors (funding, popular interest, etc.) influence publication rates for individual genes at each translational phase. Although our work establishes a baseline approach for tracking the translational trajectory of human genes into clinical and population health impact, future analyses will have to develop models of translation trajectories for specific genes and their associated diseases.

## Methods

### Collecting overall publications on human genes

To obtain the publication count specific to each gene, we collected Gene-PubMed identifier (PMID) data separately from the three databases. For publications on genes indexed in PubMed, we used the “gene2pubmed” file obtained from the NCBI gene website, as similarly done in the study of Stoeger et al.^2^ Human data (Taxonomy ID is 9606) from the “gene2pubmed” file was used here, providing gene-PMID crosslinking information, which is either manually curated by indexers from the National Library of Medicine or integrated from other public databases.

The publications in translational research T1 and T2–T4 phases were downloaded from HuGE and GPH databases, respectively. The selected articles downloaded from the GPH were previously identified as original research studies, studies on evidence synthesis, and/or guidelines publications. Excluded from GPH for this analysis were reviews, commentaries, and methods articles. The gene-PMID data from HuGE and GPH were ascertained using an automated literature annotation tool, PubTator^14,15^ PubTator provides gene-PMID information collected using automated named-entity recognition tools and third-party resources. We found genes mentioned in each abstract of the publication in HuGE and GPH, and used them to count publications for each gene.

### Gene ranking based upon number of publications

To compare the publication count on genes in HuGE and GPH to the entire literature in PubMed, we ranked the genes for those with the most publications with resulting top most common genes. Additionally, results were calculated using the *z*-score of each gene representing the significance of the publication count difference between two datasets (PubMed vs HuGE or GPH) for top percentage of genes. The statistical significance of resulting *z*-scores were calculated for all genes having equal to or greater than five publications. Longitudinal publication count data (from 2000 to 2017 for PubMed and HuGE and 2011 to 2017 for GPH) for a few selected top genes was also performed. To understand disease association of these publication changes, we also added the numbers of HLBS disorders, cancer, and rare disease related publications obtained from our former research.^16,17^

### Correlation analysis

To explore the correlation in publication count for genes between PubMed, HuGE, and GPH, we obtained Pearson correlation coefficients and Spearman’s rank correlation coefficients. We drew a linear regression line in correlation figures. Correlation coefficients, and linear regression line was performed using SciPy python library.^18^

### Reporting Summary

Further information on research design is available in the Nature Research Reporting Summary linked to this article.

## Supplementary information

Supplementary Information

Reporting Summary

## Data Availability

All data generated or analyzed during this study are publicly available at Public Health Genomics and Precision Health Knowledge Base website, PubMed FTP (ftp://ftp.ncbi.nlm.nih.gov/gene/DATA/gene2pubmed.gz) and PubTator FTP (ftp://ftp.ncbi.nlm.nih.gov/pub/lu/PubTator/). The organized data are also available as Supplementary data file.
