# Supplementary material for: Tracking human genes along the translational continuum
Source: NPJ Genom Med. 2019 Oct 16;4:25. doi: 10.1038/s41525-019-0100-0 (PMC6795796; doi:10.1038/s41525-019-0100-0)
Supplement: Supplementary file 1 — Supplementary Information [file 41525_2019_100_MOESM1_ESM.pdf]

Supplementary information file.

Supplementary Figure 1. Phases of translational research and descriptive terms along the continuum. (T0): T1, developing candidate health applications; T2, evaluating candidate health applications and developing evidence-based recommendations; T3, integrating evidence-based recommendations into care and prevention; and T4, assessing health outcomes and population impact.

Supplementary Figure 2. Venn Diagram of genes in three databases. While PubMed contains publications on 24,656 genes, only 11,081 (44.94%) and 1,846 (7.49%) genes are identified in HuGE and GPH, respectively.

Supplementary Figure 3. Comparison of publications in HuGE and GPH over PubMed. Publication count of HuGE shows more closely and positively related to the publication count of PubMed than that of GPH. Figure (b) shows that GPH focuses on a few selected genes.

Supplementary Figure 4. Publication number on select genes by years in all HuGE, all GPH, PubMed+HuGE+GPH (All-source), and subcategories of cancer, heart, lung, blood, and sleep (HLBS), and rare disorders.

Supplementary Note 1. A description of how publications are assigned to the HuGE Literature Database and the GPH Literature database follows. Additionally, the specificity of paper assignment and other evaluation measures are described.

Supplementary Table 1. Performance test results comparing SVM results with known classification in test set (data selected from PubMed during five consecutive weeks from Feb 22, 2007 to March 28, 2007)

Supplementary Data 1. Excel file with the number of publications in each database

Supplementary Figure 1. Phases of translational research and descriptive terms along the continuum. (T0): T1, developing candidate health applications; T2, evaluating candidate health applications and developing evidence-based recommendations; T3, integrating evidence-based recommendations into care and prevention; and T4, assessing health outcomes and population impact.

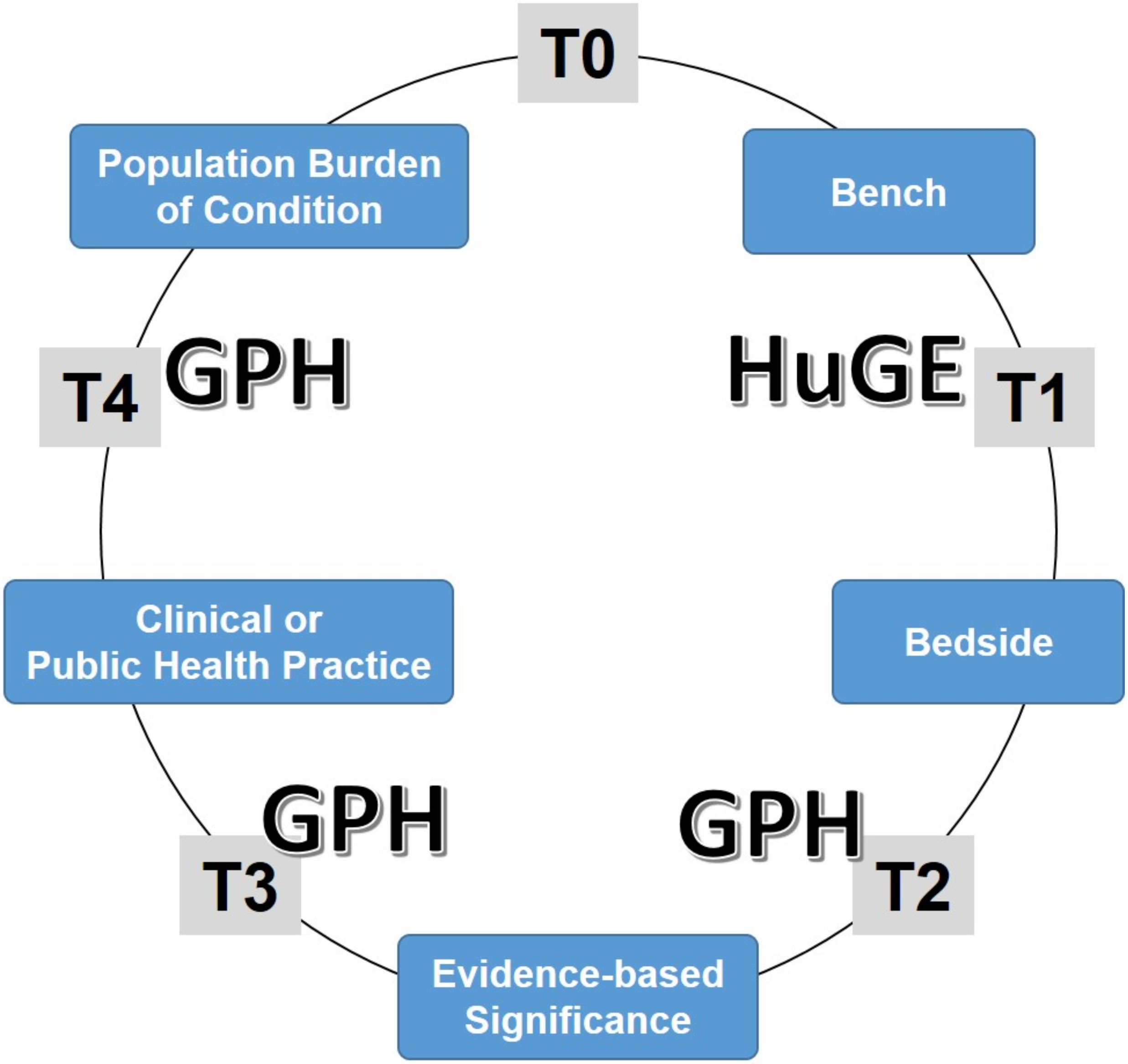

Supplementary Figure 2. Venn Diagram of genes in three databases. While PubMed contains publications on 24,656 genes, only 10,90011,081 (44.2144.94%) and 1,846 (7.49%) genes are identified in HuGE and GPH, respectively.

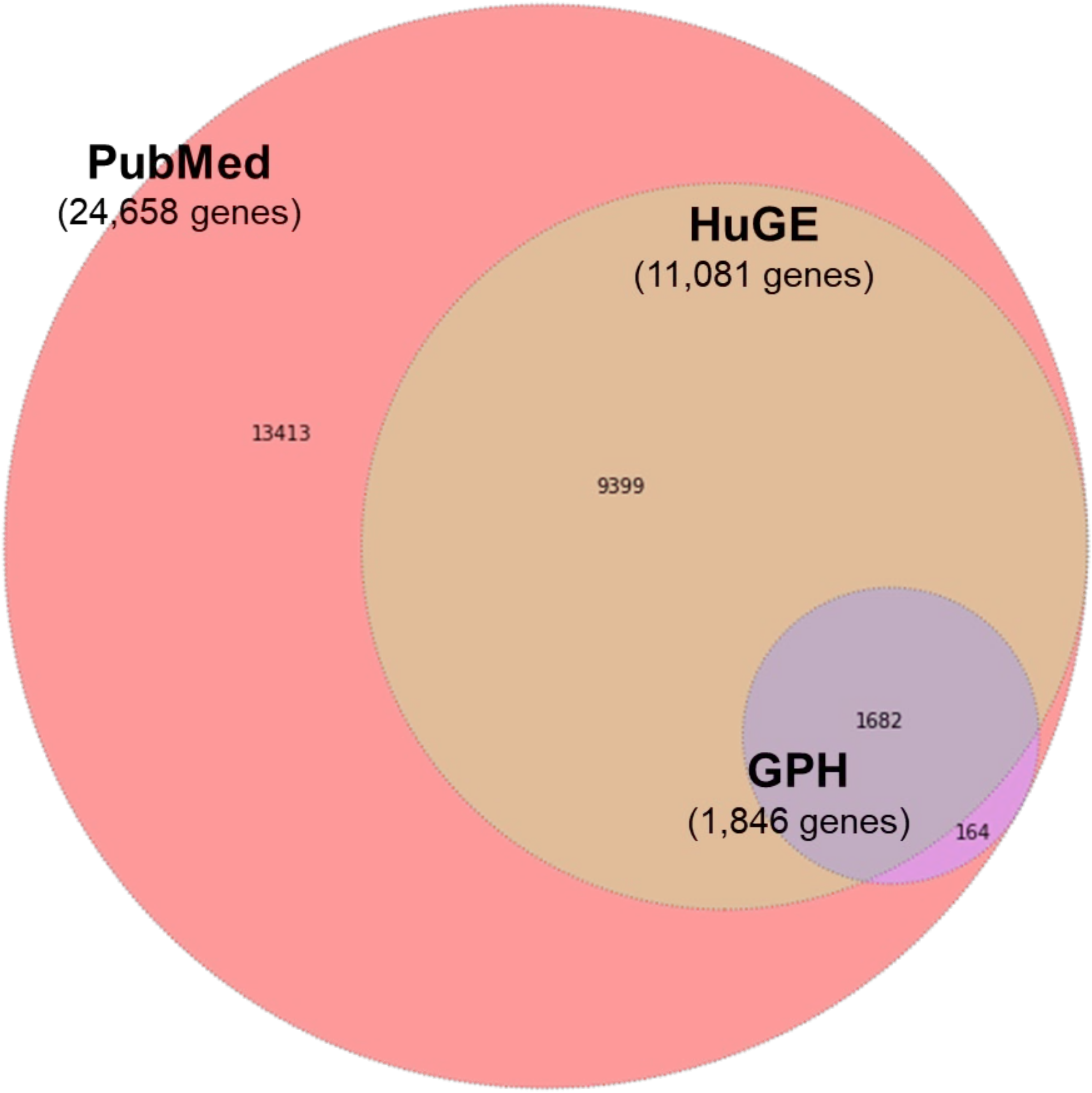

Supplementary Figure 3. Comparison of publications in HuGE and GPH over PubMed. Publication count of HuGE shows more closely and positively related to the publication count of PubMed than that of GPH. Figure (b) shows that GPH focuses on a few selected genes.

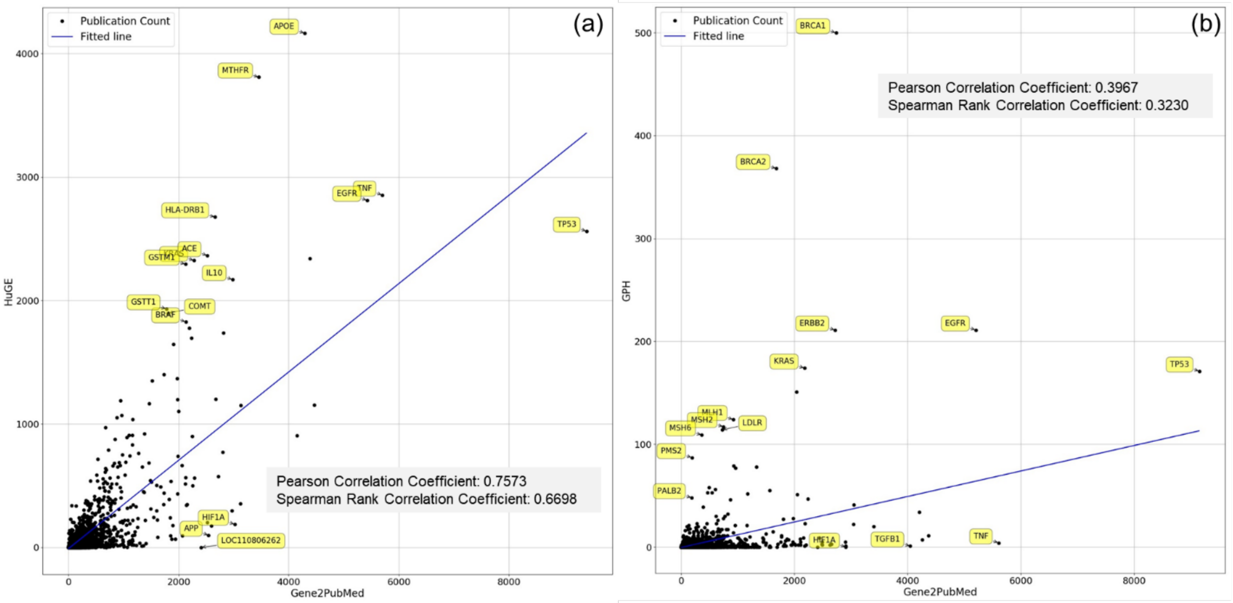

Supplementary Figure 4. Publication number on select genes by years in all HuGE, all GPH, PubMed+HuGE+GPH (All-source), and subcategories of cancer, heart, lung, blood, and sleep (HLBS), and rare disorders.

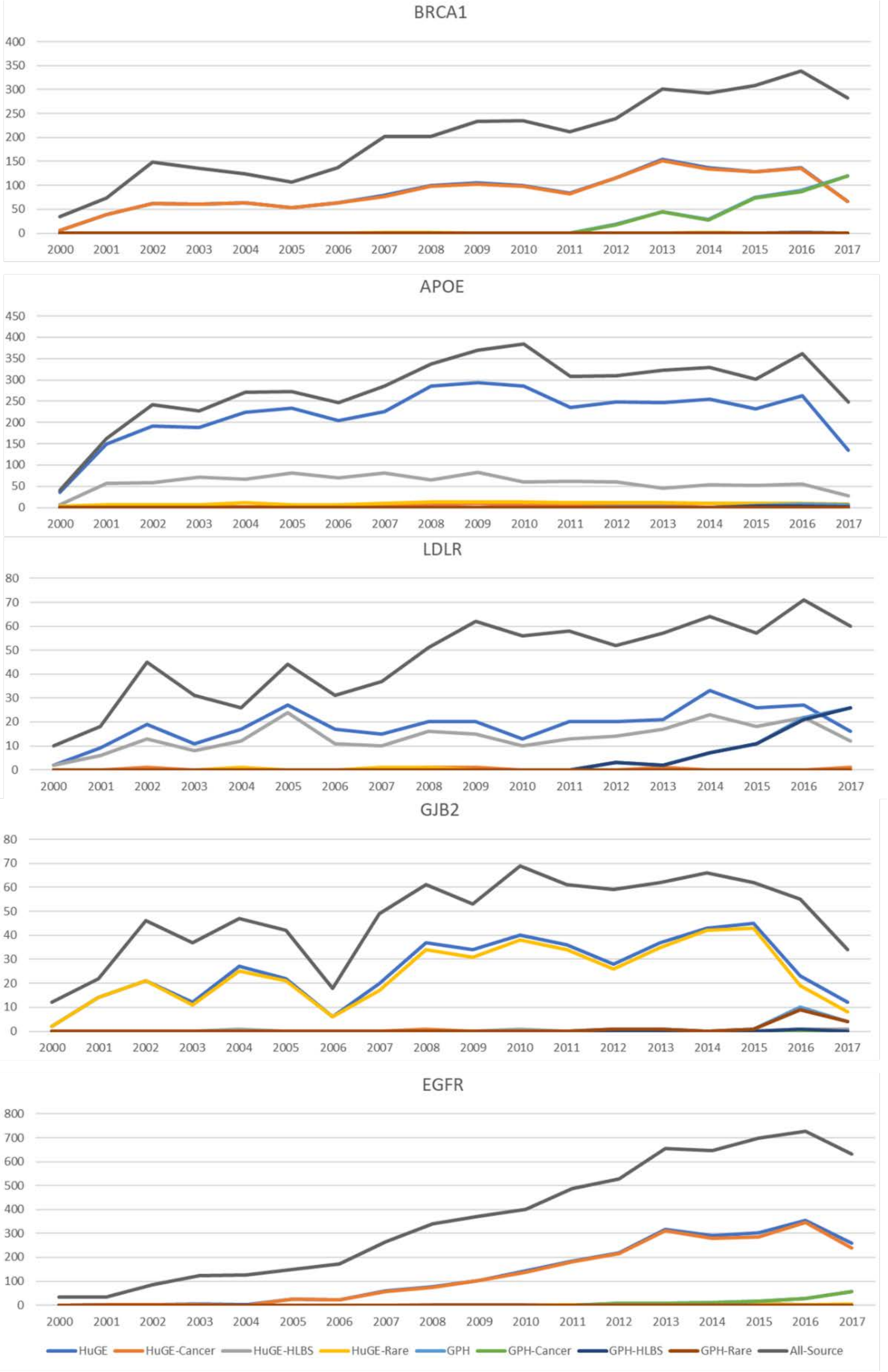

Supplementary Note 1. A description of how publications are assigned to the HuGE Literature Database and the GPH Literature database follows. Additionally, the specificity of paper assignment and other evaluation measures are described.

HuGE Literature Database

The HuGE Literature Database was originally established in 2001, which was a compilation of gene-disease association articles from PubMed with inclusion criteria of 1) articles published since October 1, 2000, 2) English-language abstract, 3) human study population, 4) genotype measured or inferred at one or more loci, 5) epidemiologic study design: cohort, case-control, case only, clinical trial, and 6) population-based analysis (genotype prevalence, association with disease, interactions with other genes or environmental factors, validity, or utility of genetic test). Articles were excluded if any of the following applied: no human data, no genotype studied, or not a population-based analysis. In 2006, sensitivity and specificity of the routine extraction process was assessed by an independent abstractor. Sensitivity was analyzed by reviewing all 4,556 articles that had been entered into PubMed on six randomly selected dates between October 2000 and December 2002. After review by two other researchers, the narrowed list included 39 eligible articles; of these, 31 had been previously identified by the routine extraction process, for a sensitivity of 80 percent. Although the search query used during that period had identified all 39 articles, eight had been incorrectly excluded by the curator. Specificity was assessed through analysis of articles included in HuGE Pub Lit to determine whether any should be excluded, and none were, for a specificity of 100 percent. (see Yu W, Gwinn M, Clyne M, Yesupriya A, Khoury MJ. A navigator for human genome epidemiology. Nat Genet. 2008 Feb;40(2):124-5.)

Beginning in 2008, the manual curation process was replaced with a new approach using machine learning (SVM), using a Java-based application named GAPscreener (Genetic Association Publication screener). To evaluate the performance of the GAPscreener, a series of new test data (not included in the training set) was used. The first test data set (92253 negatives, 773 positives) consisted of selections from PubMed during five consecutive weeks (February 22, 2007 to March 22, 2007) according to the routine, traditional screening process used to build the HuGE Navigator. Positive or negative status assigned by the routine process was considered the gold standard. We used this data set to evaluate two keyword weighting schemes. A second data set (68255 negatives, 597 positives), selected from PubMed during four subsequent weeks (April 5, 2007 to April 26, 2007), was used to evaluate false-positive results generated by the GAPscreener using the selected weighting scheme. Recall, specificity and precision were calculated from the test data to evaluate the performance of the application (data selected from PubMed during five consecutive weeks from Feb 22, 2007 to March 28, 2007). Separate logistic regression models for results of the one-way and two-way SVM schemes during the 5-week experiment were performed. Results from each model were used to generate receiver-operating characteristics (ROC) and calculate the area under the curve (AUC) with 95% confidence intervals. The AUC of ROC curves for the two models were compared using nonparametric methods (see Supplementary Table 1 and Yu W, Clyne M, Dolan SM, Yesupriya A, Wulf A, Liu T, Khoury MJ, Gwinn M. GAPscreener: an automatic tool for screening human genetic association literature in PubMed using the support vector machine technique. BMC Bioinformatics. 2008 Apr 22;9:205.).

GPH Literature Database

The GPH Literature Database contains PubMed publications on translational phases of research restricted to T2 (evidence-based applications; what works?) and (T3–T4: practice and control programs and population health disease burden; how has it been implemented and is it working in the real world?). Articles falling into T0 (scientific discovery) and T1 (candidate application) are excluded. All articles are identified through a manual targeted text query search. Although specificity has steadily increased through manual curation by multiple reviewers (not published), it is more difficult to accurately quantify the number of missed items (sensitivity). A random check within the GPH Literature Database for articles identified through other activities (e.g. highlighted in a genomic translational newsletter, or through a journal search), is performed on a continuous basis, which has not resulted in any relevant articles that have been missed.

Publications collected by this process are reviewed and classified into groups by a) original studies, b) Research Synthesis / Modeling / Meta-Analysis/ Systematic reviews / Narrative reviews, c) Guidelines / Policies / Recommendations, and d) Tools/ Methods / Training / Education / Decision Support by two or more coders. In this short report, we limit our analysis to papers categorized as a), b) and c). (see Clyne M, Schully SD, Dotson WD, Douglas MP, Gwinn M, Kolor K, Wulf A, Bowen MS, Khoury MJ. Horizon scanning for translational genomic research beyond bench to bedside. Genet Med. 2014 Jan 9.)

Supplementary Table 1. Performance test results comparing SVM results with known classification in test set (data selected from PubMed during five consecutive weeks from Feb 22, 2007 to March 28, 2007)

	Test Parameters	22-Feb-07	1-Mar-07	8-Mar-07	15-Mar-07	22-Mar-07	ROC area (95% CI)	*p* value
One Way	Recall	0.946	0.968	0.951	0.965	0.951	0.967	< 0.0001
	Precision	0.345	0.297	0.265	0.298	0.265	(0.958–0.975)	
	Specificity	0.981	0.981	0.980	0.981	0.980		
Two Way	Recall	0.946	0.992	0.967	0.977	0.993	0.982	
	Precision	0.345	0.311	0.291	0.323	0.336	(0.976 – 0.987)	
	Specificity	0.981	0.982	0.982	0.983	0.984		

One-way: key words with z scores greater than 1.96 were selected as featured key words.

Two-way: key words with z scores greater than 1.96 or less than -1.96 were selected as featured key words.

AUC: area under the curve.

CI: confident interval
